# Everybody's Page

**Published:** 1890-11-08

**Authors:** 


### Everybody's Page.

"Hay Fever may be likened to a tie vote; the eyes and nose both appear to have it."

The United States Marine Hospital Service are going to recommend the inspection of all persons immigrating to the States, which, if effected, will surely tend to diminish the numbers who cross the Atlantic.

The knowledge that "vinegar eels" exist in weak or impure vinegar may induce people to buy the best or go without. It is not known at what stage of the preparation or how they get into the vinegar, and "vinegar eels" are not dangerous, but there is something nasty and unappetising about salad and live "eels."

No better way can be found for preventing Acts of Parliament likely to be useful from becoming dead letters than the action of the Mansion House Council on the Dwellings of the Poor in disseminating leaflets setting forth the various provisions of the Housing of the Working Classes Act in the most effective and intelligible form, and in delivering lectures on the subject, as is to be done during the present winter.

Londoners must anxiously hope that the twenty metropolitan vestries and district boards which have received from the Metropolitan Public Gardens Association the public-spirited offer of £100 each to be expended in planting plane trees in suitable public thoroughfares in each district will rise to the occasion. The condition made with the offer, that the local authorities are to maintain the trees when planted, is so reasonable that no difficulties should stand in the way of accepting the offer.

Somebody of sound common-sense has solved the problem of what to do in the case of "If I had a donkey that wouldn't go," excepting that his solution applies to a horse. Some horses who will go very well with companions refuse to go at a decent pace when making a journey by their own sweet selves. This inventive genius proposes to make a large pair of spectacles, on the glasses of which a horse in full gallop is to be depicted. These spectacles are to be fastened on at a suitable distance in front of the horse's eyes, who, the inventor thinks, will strain every effort to catch up the horse which he sees galloping in front of him. Happy inventor! a splendid idea, but he doesn't know much about horses.

The New York *Medical Record* gives cases mentioned by Dr. Lydston of two schoolboys who stole apples; one was caught, the other escaped; the first was incarcerated with thieves and criminals, and by the time he was released his thieving propensities had developed into something far more serious. The second who escaped remained at school, gave up his mischievous tricks, studied law, and ultimately became a judge, and twenty-five years after the apple episode he convicted his former comrade of murder. This sounds too good to be true, but is, nevertheless, authentic. The *Record* says that the specific microbe of crime must be discovered, so that humanity shall be inoculated with the attenuated virus of crime which will purge it of bad actions. This is one way of putting it, but the story is an excellent example of the doubtful good of reformatories where the evil element is predominant. There is something to us, too, very hysterically moral in the rule laid down by many institutions that no child shall receive their benefits who has been convicted of *crime*, on the principle that a hungry child who grabs a loaf is to get no chance of moral improvement. Evil is contagious, but, then, so is good; but this fact seems scarcely recognised in the treatment of juvenile offenders.

The Jewish population of France is said to number about 130,000, of whom 50,000 live in Paris.

We are glad to hear that the scheme of the proprietors of *Education* for the establishment of field classes has met with such a hearty support from headmasters of both public and private schools, that a start has already been made by the establishment of a Geological Field Class. Any objection that might have been made by parents on the score of expense is practically met by confining the average cost of each excursion into the country to half-a-crown, including the moderate fee of one shilling for each class.

We have already had a foretaste of winter favours to come in the shape of fogs, with their accompanying gloom and depression. One form of these fogs is both mentally and physically irritating; while its particles which are coated with the soot and sulphur with which the air is impregnated are causing such exquisite tortures to the eyes, nostrils, and throat, we are painfully aware that above the fog which is confined to the lower-lying ground, the sun is shining in a brilliant sky as though mocking us for our perverseness in making no efforts to mitigate the evils against which we so inconsequently grumble. In cold weather the extra fires lighted help to aggravate the evil, but the rich, who can afford the electric light, can at least enjoy less vitiated air than those who have to fall back on gas as an illuminant.

The problems of poverty are unfortunately more easily stated than solved. The causes are as numerous as they are difficult to grapple with. The most prolific cause of all—intemperance—which is so hopelessly lowering to the physical, mental, and moral condition of the people, seems to be the hardest to contend against. In Ireland ether drinking has lately become prevalent. To the confirmed toper ether has much stronger recommendations and far greater fascinations than the much-prized "drop o' the crathur." Its special feature, which is a source of such unbounded delight to those who have a predeliction for being drunk in preference to the monotony and dryness of sobriety, is that it makes you drunk with great rapidity and its effects pass off as rapidly. Therefore the confirmed toper can get drunk and also quarrelsome, for that is one of the effects of ether, several times a day. Ether drinking may be the drunkards' paradise, but it bids fair to become society's pest unless its sale is restricted in some manner.

Mr. Josiah Oldfield has been lecturing on School Diet, which, he says, in order to be satisfactory, must be capable of repairing the wear and tear caused by mental and physical strain, it must contain a special proportion of tissue-forming elements, it must not be monotonous, it must be appetising, easily prepared, and inexpensive. Roughly speaking, the elements necessary are albuminoids, fats, carbo-hydrates, mineral matter, and water. Fish, flesh, and fowl Mr. Oldfield ignores utterly; he falls into the error of all vegetarians of evading the question that the essentials for growth may be found in flesh. He just touches on the fact that the necessary fat may be found on beef or mutton, but he remarks rather naively, "my experience of this fat in schools is that most of it is left at the side of the plate, or else finds its way to the dog." Here Mr. Oldfield distinctly scores a point, but we decline to agree with him that *corpus sanum* can be maintained on vegetables solely. We have a clinging John Bull belief in the good effect of roast beef in moderation, accompanied by vegetables.

